# Dr. Grosvenor R. Trowbridge

**Published:** 1908-06

**Authors:** 


### OBITUARY

---

Dr. Grosvenor R. Trowbridge, of Buffalo, died at his home 410 Elmwood Avenue, May 20, 1908, aged 44 years, after an illness of several weeks duration. He was the son of Dr. John S., and Abby Heacock Trowbridge, and was born in this city September

14, 1863. He was the grandson of Dr. Josiah Trowbridge, who was a pioneer in professional life in Buffalo and who was mayor of the city in 1837. He was a nephew of the Rev. Grosvenor W. Heacock, who was for a generation pastor of the Lafayette street Presbyterian Church. On his mother's side he bore relationship to the famous Grosvenor family, one of whom, Seth Grosvenor of New York, founded the Grosvenor library.

Grosvenor R. Trowbridge, as thus will be observed, was a descendant from two of Buffalo's oldest and best known families. He received his preliminary education at Dr. Brigg's school for boys, and took his academic degree from Williams College in 1884. He then entered the medical department of the University of Buffalo, from which he graduated in medicine in the class of 1887. Dr. Trowbridge then served a term as interne at the Rochester City Hospital and afterward became assistant physician at the hospital for the insane at Danville, Pa.

In 1893, Dr. Trowbridge married Lucy S., daughter of Col. Joseph H., and Abbie H.. Horton. He had practised his profession in Buffalo for the last fifteen years.

Dr. Trowbridge was a member of the American Academy of Medicine, the Buffalo Academy of Medicine, the Medical Society of the County of Erie, the Medical Union of Buffalo, the Esculapian Club and the University, Park and Transportation Clubs. He was a 32d degree Mason and a member of the Sons of the American Revolution. At the time of his death he was surgeon for the Delaware, Lackawanna & Western Railroad Company, and the Lehigh Valley Railroad Company. At one time he was president of the Lehigh Valley Surgeons' Association and he was an officer of the recently organised surgeons' association of the Delaware, Lackawanna & Western. He was on the board of the Children's Aid Society.

Dr. Trowbridge's funeral was largely attended at the family residence Saturday afternoon, May 23, and the interment was at Forest Lawn. Dr. Trowbridge is survived by his wife. He was a skilful surgeon, an excellent physician, a most amiable man, possessing the confidence and esteem of a large circle of friends.

